# Na^+^/H^+ ^exchanger regulatory factor 1 inhibits platelet-derived growth factor signaling in breast cancer cells

**DOI:** 10.1186/bcr1846

**Published:** 2008-01-11

**Authors:** Yong Pan, Edward J Weinman, Jia Le Dai

**Affiliations:** 1 Department of Molecular Pathology, The University of Texas M.D. Anderson Cancer Center, Fannin Street, Houston, Texas 77054, USA; 2 Department of Medicine, University of Maryland School of Medicine, Greene Street, Baltimore, Maryland 21201, USA; 3 Department of Physiology, University of Maryland School of Medicine, Greene Street, Baltimore, Maryland 21201, USA; 4 Medical Service, Department of Veterans Affairs Medical Center, Greene Street, Baltimore, Maryland 21201, USA; 5 The University of Texas Graduate School of Biomedical Sciences at Houston, Bertner Avenue, Houston, Texas 77030, USA

## Abstract

**Introduction:**

The gene encoding Na^+^/H^+ ^exchanger regulatory factor 1 (NHERF1) is a putative tumor suppressor gene that harbors frequent loss of heterozygosity (LOH) and intragenic mutations in breast carcinoma. The exact biologic activity of NHERF1 in mammary glands, however, remains unclear. It was recently proposed that NHERF1 forms a ternary complex with platelet-derived growth factor receptor (PDGFR) and phosphatase and tensin homolog (PTEN), linking NHERF1 suppressor activity to PDGF-initiated phosphoinositide-3 kinase (PI3K)/PTEN signaling.

**Methods:**

The effect of NHERF1 on the kinetics of PDGF-induced Akt activation was determined in cells with varied *NHERF1 *background. Levels of active Akt in mammary gland of *NHERF1 *knockout and wild-type mice were compared. We also examined how NHERF1 expression status affects cell sensitivity to PDGFR inhibitor. A plausible connection between NHERF1 and PTEN pathway was explored at the genetic level.

**Results:**

We showed that NHERF1, through its PDZ-I domain, interacts directly with the carboxyl-terminal tail of PTEN. Knocking down NHERF1 expression in Zr75.1 cells markedly delayed the turnover of PDGF-induced phospho-Akt. Conversely, NHERF1 over-expression in MCF10A cells led to accelerated phospho-Akt degradation. The slowed decay of phospho-Akt that resulted from NHERF1 loss was evident in mouse embryonic fibroblasts isolated from *NHERF1 *knockout mice. In agreement with this, mammary gland tissues from these mice exhibited markedly elevated phospho-Akt. The responses of breast cancer cells to PDGFR inhibition were also altered by changes in NHERF1 expression level. Zr75.1 cells with NHERF1 knockdown were more resistant to STI-571-induced apoptosis than parental cells. Similarly, over-expression of NHERF1 rendered MCF10A cells more sensitive to STI-571. NHERF1-induced apoptotic response relies on an intact PTEN pathway; over-expression of NHERF1 in MCF10A cells with PTEN knockdown did not affect STI-571 sensitivity. It was found that *NHERF1 *LOH-positive breast cancer cells had reduced NHERF1 expression. Interestingly, these cells more frequently had wild-type *PTEN *or *PI3KCA *gene than the LOH-negative lines.

**Conclusion:**

Our data indicate that the interaction of NHERF1 with PTEN counterbalances PI3K/Akt oncogenic signaling and may affect how cells respond to PDGFR inhibition in breast cancer. The dependence of NHERF1 responses on PTEN and genetic segregation of *NHERF1 *and *PTEN *(or *PI3KCA*) alterations suggest that NHERF1 is an active component of the PTEN pathway. Collectively, our study indicates that the biologic activity of NHERF1 in mammary gland is related to PTEN signaling.

## Introduction

The gene encoding Na^+^/H^+ ^exchanger regulatory factor 1 (NHERF1) (also known as EBP-50 or NHERF) is a candidate tumor suppressor gene in human breast cancer. Human *NHERF1 *cDNA encodes a protein of 358 amino acids in length. NHERF1 and its close homolog NHERF2 (also known as E3KARP or TKA1) share two modular structures: two tandem PDZ domains at the amino-terminus and an ezrin-radixin-moesin (ERM)-interacting domain at the carboxyl-terminus [1]. NHERF1 and NHERF2 are differentially expressed in mammalian tissues, with particularly high levels found in polarized epithelial cells [2]. NHERF1 acts as an important regulator and integrator of multiple signaling pathways by virtue of its ability to bind to a variety of proteins through its PDZ (PSD-95/Dlg/ZO1) domains and ERM-interacting domain. Via its PDZ domains, NHERF1 recognizes a carboxyl-terminal motif, D(S/T)XL, that is present in a number of transmembrane proteins, such as platelet-derived growth factor receptor (PDGFR) [3,4], cystic fibrosis transmembrane conductance regulator [5-7], β_2_-adrenergic receptor [8,9], and sodium bicarbonate co-transporter [10]. NHERF1 also interacts with a variety of intracellular proteins, including phospholipase C-β isoforms, G protein-coupled receptor kinase 6A, spleen tyrosine kinase (SYK) and Yes-associated protein 65 [11-15]. Via its carboxyl-terminal ERM-interacting domain, NHERF1 binds to ERM proteins, a family of actin cytoskeletal adaptors. One ERM family member is merlin, the product of *NF2 *tumor suppressor gene. Germline mutations of *NF2 *have been implicated in predisposition to meningiomas and schwanomas [16,17]. The amino-terminus of the ERM family proteins (ERM domain) binds to the ERM-interacting domain of NHERF1; this interaction may be important for NHERF1 functions through its connection of membrane transporters and actin cytoskeleton.

The *NHERF1 *gene is located at 17q25.1. Loss of heterozygosity (LOH) at this locus occurs in more than 50% of breast tumors [15,18-23]. However, such allelic loss is infrequent in other tumor types; LOH at the *NHERF1 *locus occurred in fewer than 10% of colorectal and pancreatic cancer lines, suggesting that *NHERF1 *is specifically targeted during breast tumorigenesis [15]. We reported three cases of *NHERF1 *intragenic mutations in a panel of breast tumors pre-screened for LOH [15]; notably, all mutations were located at conserved residues of PDZ domains or ERM-interacting domain. These tumorigenic mutations interfere with NHERF1 binding to SYK or merlin (loss-of-function), suggesting their functional relevance to breast tumor initiation or progression. *NHERF1 *LOH positively correlates with aggressive features of breast tumors, including tumor size, grade and stage, indicating that *NHERF1 *plays a critical role in mammary carcinogenesis, in which its putative suppressor activity may be haplo-insufficient.

Despite the genetic evidence available, the biologic activities of NHERF1 in mammary gland were unknown. The finding that the phosphorylation status of NHERF1 oscillated during the cell cycle implicated possible link of NHERF1 to tumor-related response [24]. Indeed, the fact that NHERF1 is a substrate for cdc2, a G_2 _to M phase cyclin-dependent kinase, suggests a role of NHERF1 in cell division [24]. Using the small interfering RNA (siRNA) method, we demonstrated increased growth of breast cancer cells when NHERF1 expression was knocked down [25]. The growth promotion effect that occurred in response to NHERF1 loss was due to an accelerated G_1 _to S transition, which was accompanied by elevated levels of cyclin E and phosphorylated Rb protein. This indicated that normal NHERF1 function may involve suppression of cell cycle progression [25].

Although the identity of the NHERF1-interacting partner responsible for the cell cycle regulatory effect remains unclear, a recent report [26] showed NHERF1 binding to the carboxyl-terminal tail (PDZ-binding motif) of phosphatase and tensin homolog (PTEN). The PDZ-binding motif of PTEN was also shown to interact with membrane-associated guanylate kinase family proteins, but the biologic significance of these bindings is not clear [27-29]. The interaction of NHERF1 with PTEN and PDGFR facilitates the formation of a ternary complex. Interestingly, this complex formation was found to offset PDGF-initiated phosphorylation of downstream targets such as Akt in mouse embryonic fibroblasts (MEFs) [26]. Activated Akt (by phosphorylation) plays a pivotal role in promoting cell survival, increasing cell invasiveness and overriding cell cycle checkpoints [30]. A possible activity of NHERF1 in counteracting the Akt pro-oncogenic pathway raises an attractive mechanism that explains NHERF1 tumor suppressor activity in mammary glands. Thus, in the present study we sought to determine whether the activity of NHERF1 is associated with a PTEN-dependent pathway in breast cells, and whether NHERF1 expressional status affects PDGF-stimulated downstream cell survival signaling as well as cell responses to PDGFR inhibition.

## Materials and methods

### Cell culture

Breast cancer cell lines MCF7, MDA-MB-468, SKBr3, T47D, ZR75.1 and immortalized mammary epithelial line MCF10A were purchased from American Type Culture Collection (Manassas, VA, USA). All cell lines were cultured in recommended media.

Cultured Zr75.1, MCF10A and MEF cells were incubated with serum-free media for 1 day. They were then treated with PDGF-BB (0.5 ng/ml; Millipore, Billerica, MA, USA) for 0 to 120 minutes before cells were harvested in 1× SDS sample buffer.

### *NHERF1 *knockout mice

*NHERF1*^+/- ^mice [31] were inbred to generate littermates of three genotypes. Duplex PCR was used to genotype *NHERF1 *knockout mice. A common forward primer (5'-ctctgtttattcccagaagga-3') was included in the PCR reaction, together with reverse primers for knockout (5'-caagctcttcagcaatatcac-3') and wild-type (5'-ggttctaccagacggataaac-3') genotypes that were expected to yield 2.4-kilobase and 1.4-kilobase products, respectively. PCR conditions were described previously [15].

To harvest mammary gland of *NHERF1 *knockout mice, 10-week-old female littermates were killed by carbon dioxide inhalation. Mammary glands were collected and snap frozen at -80°C until use. Tissues were then ground while frozen in liquid nitrogen and lysed in 1× SDS sample buffer. The lysates were then subjected to immunoblotting.

To obtain MEF cells of varied *NHERF1 *genetic backgrounds, the *NHERF1*^+/- ^mice were inbred. At embryonic days 12 to 14, the embryos were dissected to remove heads and internal organs. The remaining tissues were minced, trypsinized and plated in Dulbecco's medified Eagle's medium supplemented with 10% fetal bovine serum (Invitrogen, Carlsbad, CA, USA). Genomic DNA was also harvested from MEF cells for genotyping. Early-passage MEF cells were then subjected to experimental treatment.

### DNA constructs

To prepare recombinant retrovirus to knockdown NHERF1 expression, an siRNA sequence that targeted *NHERF1 *transcript was inserted into pBabe-U6-Puro (a gift from Dr Jinsong Liu, M.D. Anderson Cancer Center, Houston, TX, USA). Two oligonucleotides (5'-ggaaactgacgagttcttcaaagctttgaagaactcgtcagtttccctttttg-3' and 5'-aattcaaaaagggaaactgacgagttcttcaaagctttgaagaactcgtcagtttcc-3') were annealed and subcloned into pBabe-U6, creating pBabe-U6/NHERF-910-Puro. Similarly, oligonuclotides (5'-ggacgaactggtgtaatgatatgaagcttcatatcattacaccagttcgtccctttttg-3' and 5'-aattcaaaaagggacgaactggtgtaatgatatgaagcttcatatcattacaccagttcgtcc-3') were used to knock down PTEN expression (pBabe-U6/PTEN-Puro).

GST-PTEN, pcDNA3.1-PTEN, and pcDNA3.1-PTEN-C124S were gifts from Dr Charles Sawyer (University of California, Los Angeles, CA, USA). To make a PTEN construct with deletion of the last six residues (TQITKV), PCR was conducted to introduce premature termination, using pcDNA3.1-PTEN as a template. This procedure created pcDNA3.1-PTEN-ΔC.

*NHERF1 *cDNA was obtained by reverse transcription from mRNA of a breast cancer cell line, SKBr3, and amplified by PCR. Reverse transcription PCR products were subcloned into a TA cloning vector (Invitrogen). *NHERF1 *cDNA was then subcloned into pcDNA3.1(+) (Invitrogen), generating pDNA3.1-NHERF1. *NHERF1 *cDNA was then enzymatically released from pcDNA3.1-NHERF1 and subcloned into pBabe-Puro, pBabe-Neo (Addgene, Cambridge, MA, USA), and pGEX2TK (GE Healthcare, Piscataway, NJ, USA), yielding pBabe-NHERF1-Puro, pBabe-NHERF1-Neo, and pGEX2TK-NHERF1, respectively. cDNA fragments encoding PDZ-I (residues 1 to 150), PDZ-II (residues 97 to 239), PDZ-I&II (residues 1 to 239), and carboxyl-terminus (residues 231–358) of NHERF1 were PCR-amplified and constructed to pcDNA3.1 or pGEX2TK. All constructs generated by PCR were verified by automated DNA sequencing.

### Retroviral infection

Retroviral stocks were made by transfecting packaging cells (amphotropic Phoenix cells) with retroviral constructs (pBabe-U6-Puro, pBabe-U6-NHERF-910-Puro, pBabe-U6-PTEN-Puro, pBabe-Puro, pBabe-NHERF1-Puro, pBabe-Neo, and pBabe-NHERF1-Neo), using Fugene 6 (Roche Applied Science, Indianapolis, IN, USA).

To prepare MCF10A cells over-expressing NHERF1, MCF10A cells were infected with NHERF1-Puro (or NHERF1-Neo for PTEN knockdown cells) recombinant retrovirus. Infected cells were selected by adding 1 to 5 μg/ml puromycin (or 600 μg/ml G418) to culture media. Surviving cells were assessed for NHERF1 expression by immunoblotting. MCF10A-NHERF1-Neo cells were further infected with PTEN-siRNA retrovirus to knockdown PTEN expression, which was determined by PTEN immunoblotting.

The expression of NHERF1 in Zr75.1 and MDA-MB-468 cells was knocked down by using retrovirus-based siRNA method. Zr75.1 cells were infected with Babe-U6-NHERF-910-Puro retrovirus and subjected to puromycin (2 μg/ml) selection. NHERF1 expression was determined by immunoblotting.

### Glutathione S-transferase pull-down assays

Glutathione S-transferase (GST) pull-down assays were used to assess the interaction of NHERF1 and PTEN. GST-PTEN or GST-NHERF1 and their derivatives were induced by isopropyl-β-D-1-thiogalactopyranoside in pGEX2TK-transformed BL21 strain and purified with glutathione-Sepharose beads (GE Healthcare).

Radio-labeled full-length or defined segments of NHERF1 and full-length or truncated PTEN were synthesized by *in vitro *transcription from pcDNA3.1 plasmids containing the *NHERF1 *or *PTEN *cDNA and translated in the presence of [^35^S]methionine (T7 Quick TNT kit; Promega, Madison, WI, USA). The translation products were mixed with purified GST-PTEN or GST-NHERF1 immobilized on the beads. Pull-down assays were performed at 4°C for 1 hour in 1× binding buffer (20 mmol/l Tris [pH 7.5], 150 mmol/l NaCl, and 1% NP-40). The beads were then washed thoroughly with 1× binding buffer. Bound proteins were eluted by boiling in 1× SDS sample buffer, separated by SDS-PAGE. Ten per cent of the input TNT lysates was also run on the PAGE to determine relative binding capacity. The PAGE gel was then dried and exposed for autoradiography.

GST pull-down assay was also used to determine whether endogenous PTEN or NHERF1 binds to recombinant NHERF1 or PTEN, respectively. MDA-MB-468, MCF7, and T47D cells were harvested by adding 1× lysis buffer (50 mmol/l Tris-HCl [pH 7.5], 150 mmol/l NaCl, 1 mmol/l EDTA, 1% Triton-X 100, 10 mmol/l NaF, 1 mmol/l Na_3_VO_4 _and 1× protease inhibitor cocktail [Sigma, St. Louis, MO, USA]). The cell lysates were then incubated with beads coated with GST fusion proteins. The incubation lasted for 2 hours at 4°C. The beads were then washed thoroughly with 1× binding buffer before being boiled in 1× SDS sample buffer. The eluted proteins were then subjected to NHERF1 or PTEN immunoblotting.

### Immunoprecipitation and immunoblotting

To assess the interaction of PTEN with NHERF1 at the endogenous level, cultured MCF7 or Zr75.1 cells were lysed with 1× NETN buffer (20 mmol/l Tris-HCl [pH 7.5], 150 mmol/l NaCl, 1 mmol/l EDTA, 0.5% NP-40, 30 μg/ml aprotinin, 5 mmol/l PMSF, 25 mmol/l NaF, and 2 mmol/l Na_3_VO_4_). The soluble proteins (about 600 μg) were immunoprecipitated with 2 μg of goat IgG reactive to PTEN (N-19; Santa Cruz Biotechnologies, Santa Cruz, CA, USA) at 4°C overnight, using normal goat IgG as a control. The immunocomplex was collected by addition of 50 μl of agarose-conjugated protein G (Roche Applied Science) and detected with anti-NHERF1 antibody.

Protein concentrations were measured by using BCA reagent. Lysates were then subjected to immunodetection of phospho-Akt (p-Akt) and total Akt. Immunoblottings were carried out essentially as described previously [32]. Antibodies used were human NHERF1 (EXBIO Praha, Vestec, Czech Republic), β-actin (Santa Cruz Biotechnology), mouse NHERF1 (Affinity Bioreagents, Golden, CO, USA), PTEN (Millipore), caspase-3, phospho-Akt-Ser473, total Akt, and phospho-p70 S6 kinase-Thr421/Ser424 (Cell Signaling Technology, Danvers, MA, USA).

### MTT assays

MTT (3-[4,5-dimethylthiazol-2-yl]-2,5-diphenyltetrazolium bromide) assays were used to measure cell viability. Cells were seeded in 96-well cluster dishes at 5,000 cells/well with 100 μl complete medium. After overnight incubation, medium was replaced to include various concentrations of STI-571 (Novartis Pharmaceuticals). After 2 days of treatment, cells were fed 100 μl fresh medium that contained 1 mg/ml MTT (Sigma). The incubation lasted for 2 hours before the medium was removed and cells dissolved in 150 μl dimethyl sulfoxide. Absorbance was measured using a multiSkan plate reader (LabSystems, Waltham, MA, USA) at a wavelength of 570 nm. Each sample was processed in triplicate. Experiments were repeated at least three times.

## Results

### Interaction of NHERF1 and PTEN

**Figure 1:**
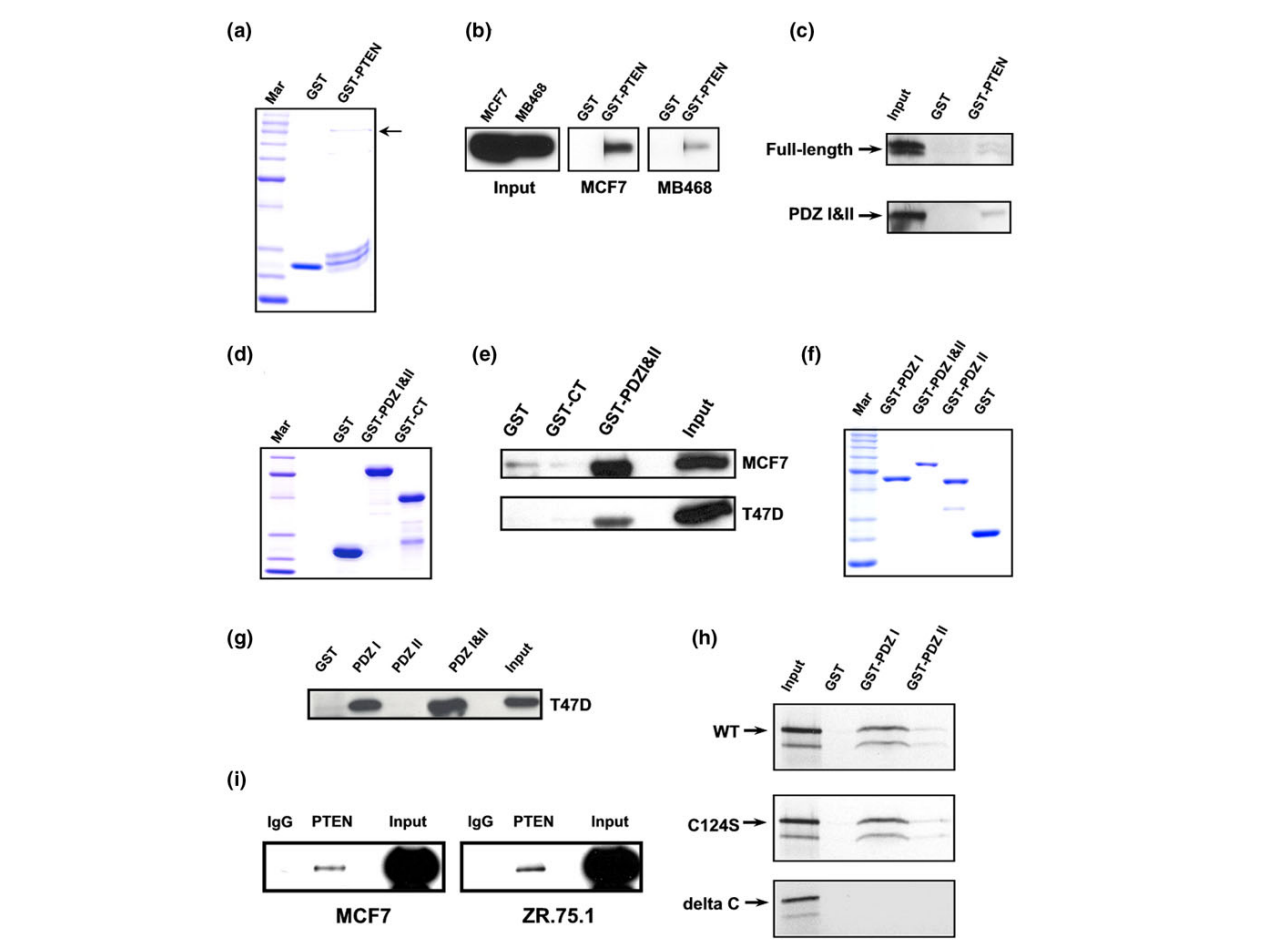
The PDZ-binding motif of PTEN associates with the PDZ-I domain of NHERF1. **(a) **Coomassie-blue staining of glutathione S-transferase (GST)-phosphatase and tensin homolog (PTEN) fusion (arrow) and GST control protein. **(b) **GST pull-down assays. GST-PTEN beads were incubated with MCF7 or MDA-MB-468 cell lysates. The protein bound on beads was separated by SDS-PAGE and subjected to Na^+^/H^+ ^exchanger regulatory factor 1 (NHERF1) immunoblotting. Ten per cent of the initial protein input was run side-by-side. **(c) **GST pull-down. The full-length NHERF1 or the PDZ-I&II domain protein was synthesized by TNT reaction in the presence of [^35^S]methionine. The labeled proteins were then incubated with GST or GST-PTEN. Components bound to GST fusion were separated by SDS-PAGE and detected by autoradiography. **(d) **Coomassie-blue staining of GST fusion of PDZ-I&II or the carboxyl-terminal half (CT) of NHERF1. **(e) **GST pull-down. Procedures were the same as those described for panel b except that MCF7 and T47D cells lysates, GST-PDZ-I&II and GST-CT were used in the reaction. Bound protein was detected by PTEN immunoblotting. **(f) **Coomassie-blue staining of GST fusion of PDZ-I or PDZ-II compared with that of PDZ-I&II. **(g) **NHERF1 interaction with PTEN through its PDZ-I domain. The experimental method was the same as that described for panel e except for the use of GST-PDZ-I and GST-PDZ-II. **(h) **PTEN binding to NHERF1 through its carboxyl-terminal PDZ-binding motif. ^35^S-labeled wild-type (WT) PTEN, a tumorigenic mutant (C124S), or a PTEN variant with deletion of the PDZ motif (delta C) were made by TNT reaction and evaluated for their binding to PDZ-I or PDZ-II of NHERF1. **(i) **Lysates from MCF7 and Zr75.1 cells were subjected to immunoprecipitation using anti-PTEN antibody or normal goat IgG. The NHERF1 protein was detected in the precipitated materials by immunoblotting. Twenty per cent of input lysates was run side-by-side.

To verify that there is an interaction between NHERF1 and PTEN, we first used GST-PTEN (Figure 1a) to pull down NHERF1 from lysates of MCF7 and MDA-MB-468 cells. We found GST-PTEN, but not GST alone, to be associated with NHERF1 (Figure 1b). Of note, the quantity of full-length GST-PTEN bound to the beads was much lower than that of the GST control (Figure 1a). As a result, much less GST-PTEN was used in the experiments. To determine whether the interaction between NHERF1 and PTEN was direct, we conducted a GST pull-down assay using *in vitro *synthesized full-length NHERF1. GST-PTEN but not GST interacted directly with the full-length NHERF1 and the PDZ-I&II domains (Figure 1c). Thus, the PDZ-I&II domains of NHERF1 are likely to mediate this direct interaction. In reverse experiments, we used GST fusion of PDZ-I and -II domains (residues 1 to 239) or carboxyl-terminal half (CT; residues 231 to 358) of NHERF1 to pull down PTEN (Figure 1d). As shown in Figure 1e, GST-PDZ-I&II bound to PTEN from MCF7 and T47D cell lysates. In contrast, CT did not bind to PTEN, confirming that NHERF1 interacted with PTEN through its PDZ domains. To further assess whether the PDZ-I or the PDZ-II domain was responsible for PTEN association, we conducted a similar GST pull-down assay using GST-PDZ-I (residues 1 to 150) or GST-PDZ-II (residues 97 to 239) beads (Figure 1f). Although PDZ-II did not interact with PTEN, PDZ-I associated with PTEN at a level similar to that of PDZ-I&II, indicating that PDZ-I is responsible for the interaction of NHERF1 with PTEN (Figure 1g).

The direct interaction between PTEN and NHERF1 was also confirmed by pull-down assays using GST-PDZ and [^35^S]-labeled PTEN products. Consistent with earlier findings, the wild-type PTEN bound strongly to PDZ-I but very weakly to PDZ-II (Figure 1h). The interaction was not affected by a tumor-derived mutation (C124S). To determine whether the PDZ-binding motif in PTEN is required for its interaction with NHERF1, we made PTEN with a deletion of the last six amino acids (ΔC). Pull-down assays, showing this PTEN mutant to be incapable of interacting with NHERF1 (Figure 1h), indicated that the PDZ-binding motif of PTEN is responsible for its binding to NHERF1. We further examined whether NHERF1 bound to PTEN at the endogenous expression level by using MCF7 and Zr75.1 cells that expressed high levels of NHERF1 and PTEN. Co-immunoprecipitation identified a significant interaction between the two proteins, demonstrating that NHERF1 associates with PTEN *in vivo *when they are expressed at the endogenous level (Figure 1i).

### NHERF1 affects cell responses to PDGF-initiated Akt phosphorylation

**Figure 2:**
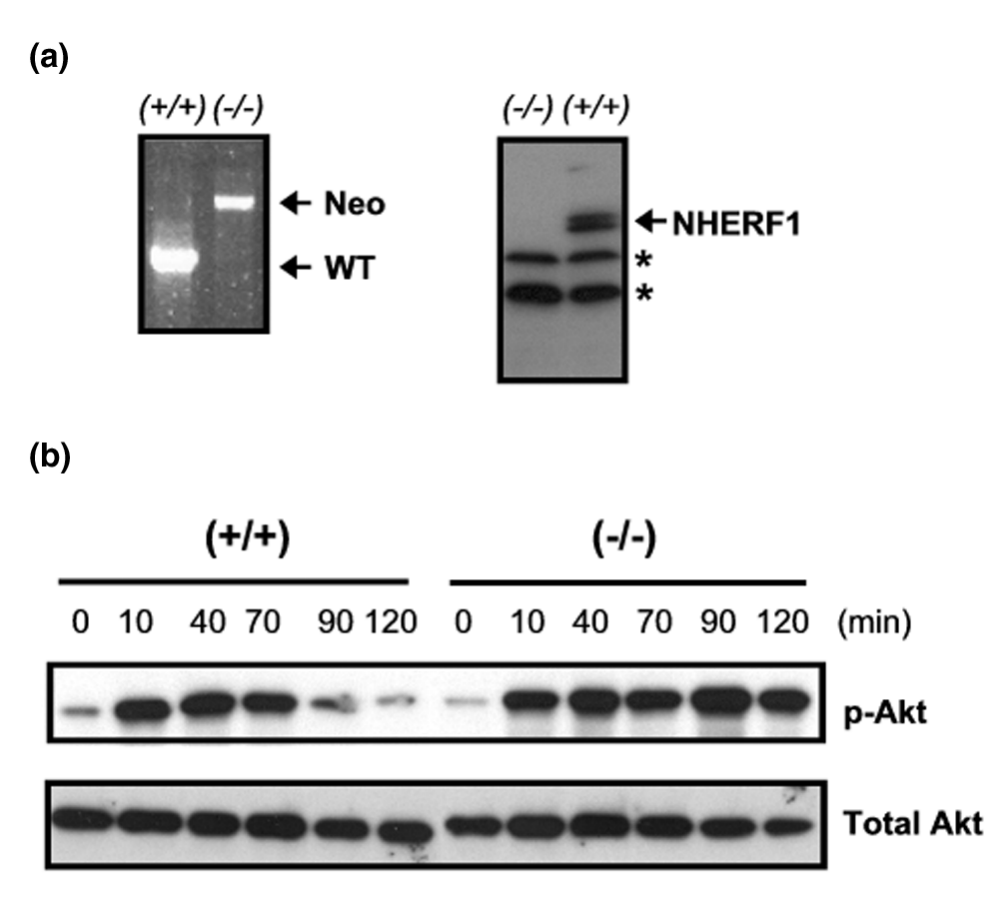
NHERF1 deletion leads to increased stability of PDGF-stimulated p-Akt signal in mouse embryonic fibroblasts. **(a) **Genotyping and Na^+^/H^+ ^exchanger regulatory factor 1 (NHERF1) expression of mouse embryonic fibroblast (MEF) cells with *NHERF1*^+/+ ^and *NHERF1*^-/- ^backgrounds. Left panel: duplex PCR was conducted to evaluate the genotypes, using the genomic DNA extracted from MEFs. Wild-type (WT) and knockout (Neo) alleles were expected to generate 1.4-kilobase and 2.4-kilobase products, respectively. Predicted genotypes are indicated. Right panel: NHERF1 expression in MEFs as measured by immunoblotting. Asterisks indicate bands from nonspecific reactivity to mouse NHERF1 antibody. **(b) **MEFs (*NHERF1*^+/+ ^or *NHERF1*^-/-^) were serum starved and stimulated with platelet-derived grwoth factor-BB (0.5 ng/ml) for indicated periods before cells were harvested for phospho-Akt (p-Akt; Ser473) measurement. Total Akt was also measured to verify equivalent loading.

**Figure 3:**
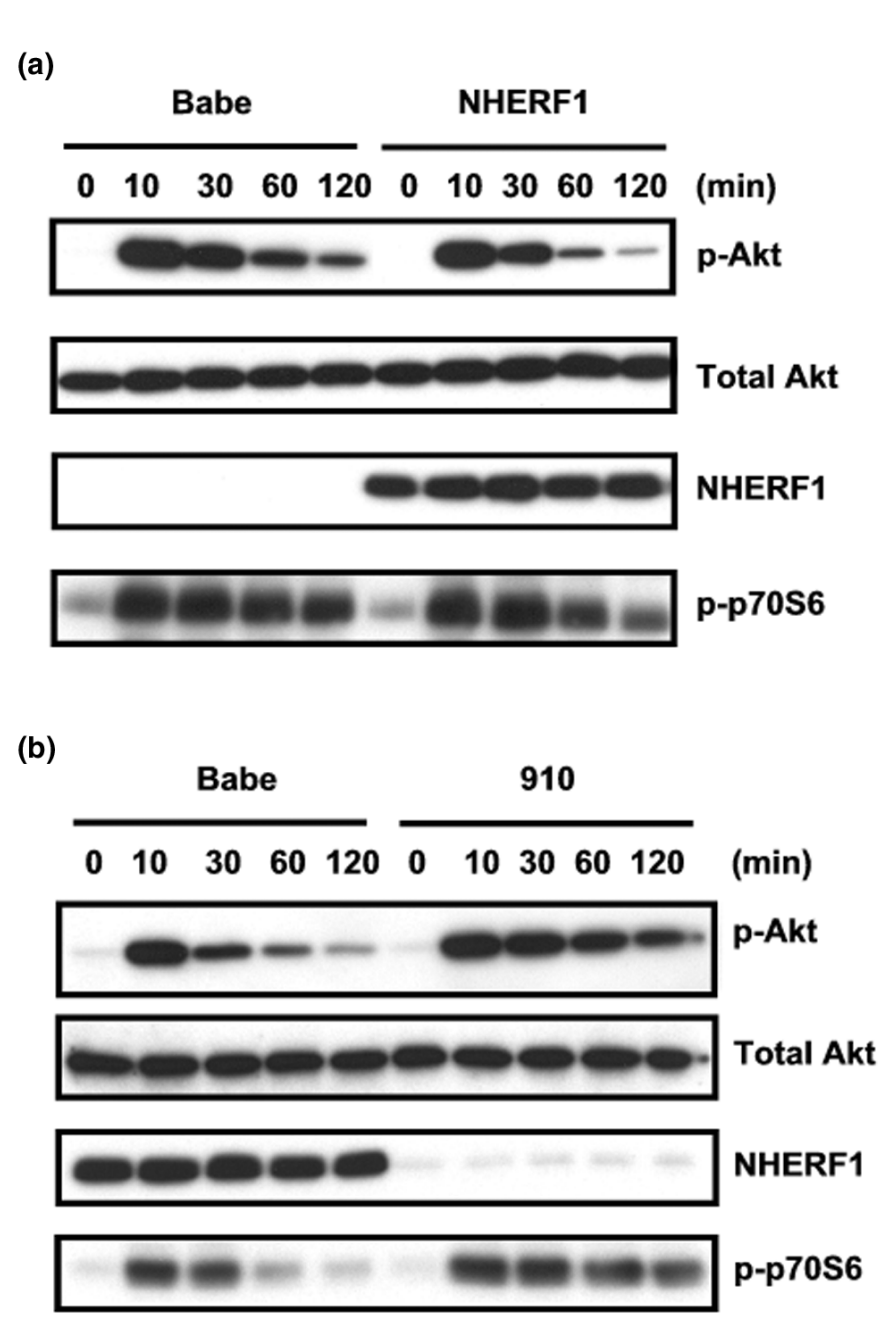
NHERF1 accelerates decay of p-Akt induced by PDGF. **(a) **MCF10A cells over-expressing Na^+^/H^+ ^exchanger regulatory factor 1 (NHERF1) or control vector (Babe) were serum starved and restimulated with platelet-derived growth factor for 0 to 120 minutes as indicated. Cells were lysed for measurement of phospho-Akt (p-Akt) level by immunoblotting. Expression status of NHERF1 was verified. Total Akt was also determined to normalize loading. **(b) **Zr75.1 cells infected with NHERF1 knockdown vector (910) or control (Babe) were analyzed as described for panel a.

Expression of NHERF1 reportedly accelerates the decay of p-Akt induced by PDGF stimulation. We first verified this conclusion in *NHERF1*^+/+ ^and *NHERF1*^-/- ^MEFs isolated from 14-day embryos that resulted from crossing of *NHERF1*^+/- ^mice. Their *NHERF1 *genetic status was determined by genotyping, and their NHERF1 protein expression was verified by immunoblotting (Figure 2a). *NHERF1*^-/- ^and *NHERF1*^+/+ ^MEFs were then subjected to treatment with PDGF ligands and harvested after 0 to120 minutes. PDGF initially induced p-Akt to similar levels in the two groups of MEFs (10 minutes). p-Akt in *NHERF1*^-/- ^cells remained at high levels at 90 and 120 minutes, but the p-Akt level in *NHERF1*^+/+ ^cells deminished markedly (Figure 2b). We repeated these experiments in two cell lines of breast origin. MCF10A cells over-expressing *NHERF1 *cDNA by retroviral infection exhibited a faster turnover of p-Akt than did control cells (Figure 3a). Likewise, knockdown of NHERF1 expression in Zr75.1 cells by retroviral infection with NHERF-910 siRNA decelerated the decay of p-Akt signals induced by PDGF (Figure 3b). In agreement with these findings, the phosphorylation status of an Akt downsream target, namely p70 S6 kinase, was consistent with p-Akt level in both MCF10A and Zr75.1 cells. These results agree with the working model that NHERF1 attenuates PDGF-initiated downstream survival signals by forming protein complex with PTEN and PDGFR.

### Higher level of p-Akt in mammary tissues of *NHERF1 *knockout mice

**Figure 4:**
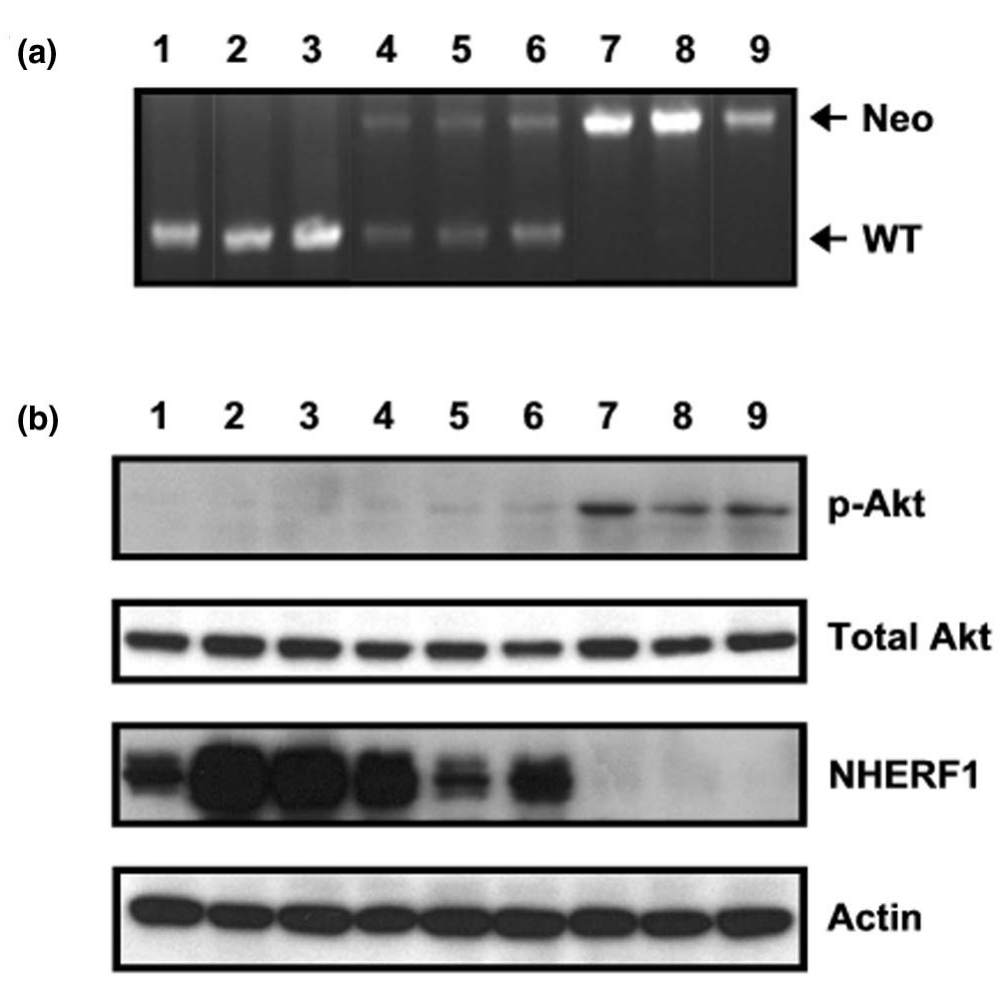
Elevated phospho-Akt levels in mammary gland of *NHERF1 *knockout mice. **(a) **Genotyping of *NHERF1 *knockout mice. Numbers 1 to 3, 4 to 6, and 7 to 9 represent *NHERF1*^+/+^, *NHERF1*^+/-^, and *NHERF1*^-/- ^mice, respectively. **(b) **Immunodetection of NHERF1 and phospho-Akt (p-Akt; Ser473) expression in mammary gland tissues from mice of respective genotypes. Membrane was stripped and reprobed with β-actin to normalize loading. WT, wild-type.

Knockout or knockdown of NHERF1 resulted in slower dephosphorylation of Akt *in vitro *(Figures 2 and 3). If this occurs *in vivo *as well, then an increase in p-Akt level would be expected in the *NHERF1 *knockout mice. To clarify this, we measured the p-Akt level in mammary gland specimens taken from the three groups of mice (*NHERF1*^+/+^, *NHERF1*^+/-^, and *NHERF1*^-/-^), whose *NHERF1 *genetic background was verified (Figure 4a). NHERF1 protein was also measured. Despite expressional variability among mice in the same group, *NHERF1*^+/- ^mice had an overall lower NHERF1 protein level in mammary glands than did wild-type mice. No NHERF1 protein was detectable in the *NHERF1*^-/- ^mice (Figure 4b). This finding was consistent with the NHERF1 expression pattern in kidney extracts among the three genotypes [31]. When p-Akt was measured, the *NHERF1*^-/- ^mammary gland was shown to maintain a markedly higher p-Akt level in comparison with that of the *NHERF1*^+/+ ^specimens, whereas the total Akt level remained little changed (Figure 4b). This result suggests that normal NHERF1 function involves suppression of Akt survival signaling by affecting the balance between PI3K and PTEN. Interestingly, compared with the wild-type mice, the *NHERF1*^+/- ^mice exhibited a moderate but consistent increase in p-Akt level (Figure 4b), indicating that decreased NHERF1 expression resulting from deletion of a single *NHERF1 *allele may be sufficient to promote cell survival in the mammary gland.

### NHERF1 affects cell sensitivity to PDGFR inhibitor

**Figure 5:**
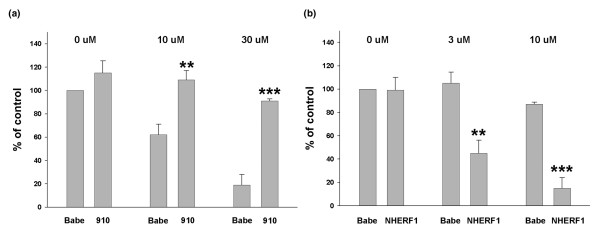
NHERF1 increases cell sensitivity to STI-571. **(a) **Zr75.1 cells with NHERF1 knockdown (910) or Babe controls were cultured in 96-well plates and treated with STI-571 (10 and 30 μmol/l) for 2 days. **(b) **MCF10A cells over-expressing Na^+^/H^+ ^exchanger regulatory factor 1 (NHERF1) or vector control were subjected to STI-571 treatment (3 and 10 μmol/l) for 2 days. MTT reagents were added to assess the relative number of surviving cells. The numbers of untreated Babe control were arbitrarily set at 100%. Data from five independent experiments are presented. **P < 0.01, ***P < 0.001 versus Babe control of the corresponding treatment.

Because NHERF1 markedly affected p-Akt turnover in response to PDGF stimulation, we surmised that NHERF1 expression status might also affect cell response to PDGFR inhibition. Zr75.1 cells with or without NHERF1 knockdown were treated with STI-571 for 2 days before MTT assay for surviving cells. STI-571 is a small molecule inhibitor of tyrosine kinases, including BCR-Abl, PDGFR and c-kit, and as a result it has been used effectively in patients with chronic myelogenous leukemia, gastrointestinal stromal tumor, and other types of tumors [33-35]. We first compared the Zr75.1 cells (base control and NHERF1 knockdown cells) in tems of their sensitivity to STI-571. While untreated, NHERF1 knockdown cells exhibited a modest increase in cell proliferation after 2 days of culture, which is in agreement with our earlier report [25]. When cells were treated with STI-571, we found that Zr75.1 cells with NHERF1 knockdown were much more resistant to STI-571 than the control (Figure 5a). Similarly, over-expression of NHERF1 in MCF10A cells rendered them more sensitive to STI-571 treatment than their corresponding Babe control (Figure 5b). These findings suggest that changes in cell survival signaling resulting from NHERF1 loss affect cell sensitivity to PDGFR blocker.

**Figure 6:**
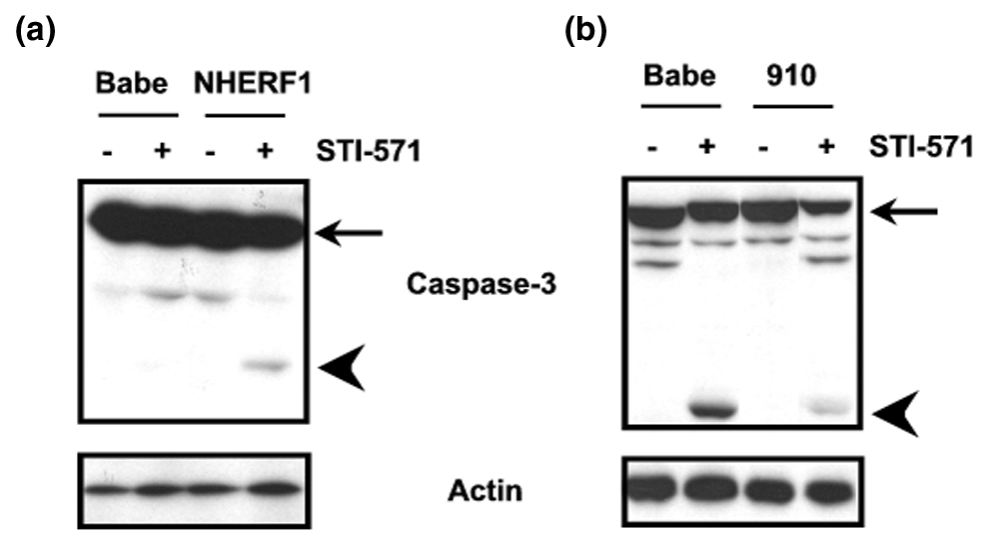
NHERF1 increases caspase-3 cleavage in response to STI-571. Cultured **(a) **MCF10A and **(b) **Zr75.1 cells of varying Na^+^/H^+ ^exchanger regulatory factor 1 (NHERF1) expression level were treated with STI-571 for 1 day. Cells were then lysed and separated by SDS-PAGE. Immunoblotting was used to detect caspase-3 (8G10). The caspase-3 antibody recognized both the full-length (35 kDa [arrow]) and the large fragment (17/19 kDa [arrowhead]) of caspase-3 resulting from cleavage.

To determine whether apoptosis is involved in the cell density difference between NHERF1-positive and NHERF1-negative cells, we harvested the cells during STI-571 treatment and assayed cell lysates for cleaved forms of caspase-3, a marker of active apoptotic process. Immunoblottings showed that before STI-571 treatment, no cleaved caspase-3 isoforms were present in either the NHERF1 knock-down or the control Zr75.1 (Babe) cells. One day of STI-571 treatment resulted in significant increases in caspase-3 cleavage in Zr75.1 Babe control cells, suggesting that apoptosis is at least partially responsible for STI-571-induced growth inhibition. Interestingly, the same treatment led to markedly lower caspase-3 cleavage in NHERF1-knockdown Zr75.1 cells (Figure 6a), which is consistent with a decreased response to STI-571-induced cell death in comparison with that of NHERF1-positive Zr75.1 cells (Figure 5a). Similar experiments were carried out on MCF10A cells, in which we showed that over-expression of NHERF1 markedly enhanced STI-571-induced cleavage of caspase-3 (Figure 6b). All of these findings indicated that loss of NHERF1 may affect cell response to growth factor inhibition as a result of increases in intrinsic cell survival [36].

**Figure 7:**
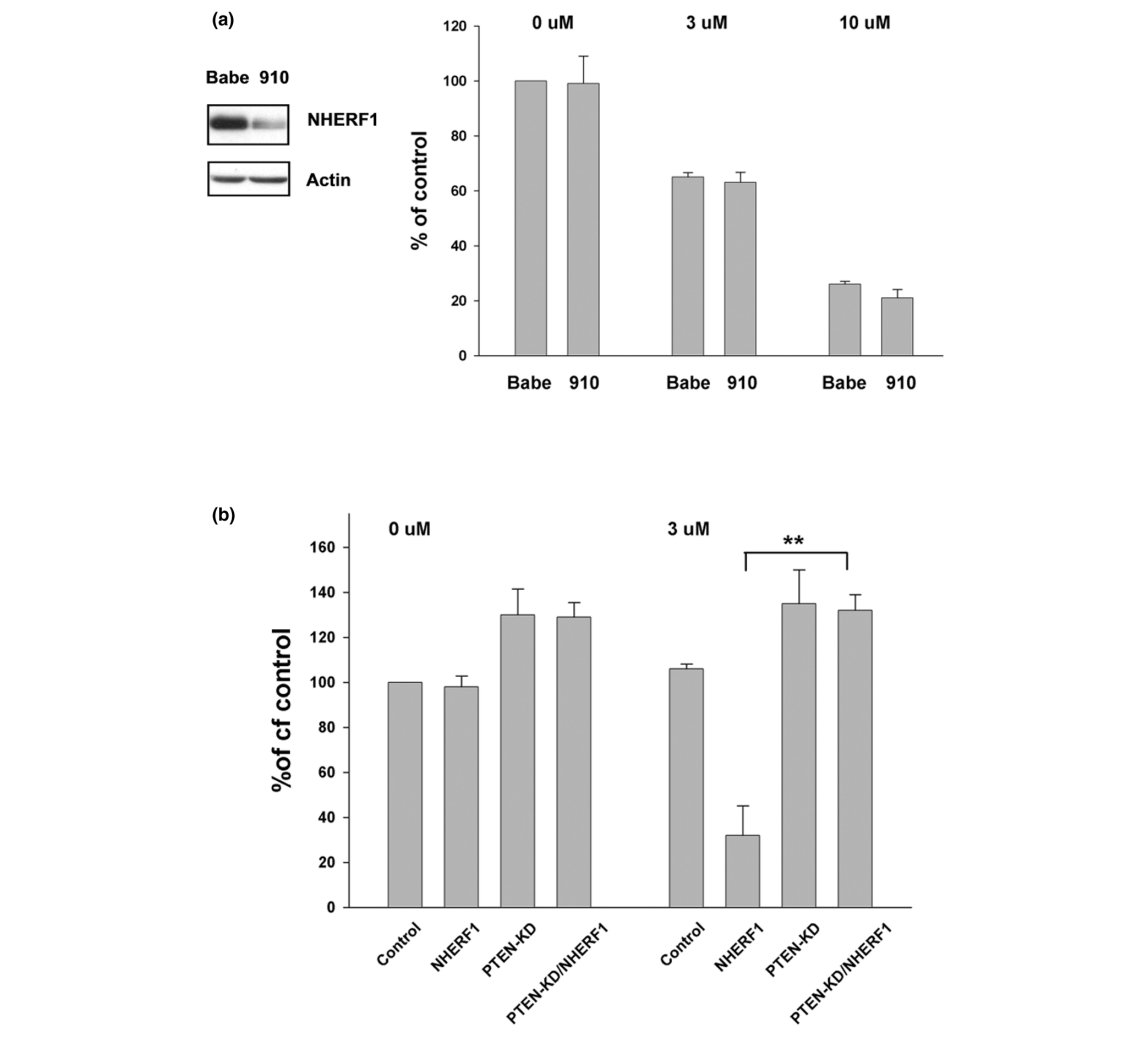
PTEN is required for NHERF1-induced increase in sensitivity to STI-571. **(a) **Phosphatase and tensin homolog (PTEN)-negative MDA-MB-468 cells were infected with Na^+^/H^+ ^exchanger regulatory factor 1 (NHERF1) small interfering RNA (siRNA; 910) or control (Babe) retrovirus. Their NHERF1 expression was measured by immunoblotting. Theses cells were treated with STI-571 (3 and 10 μmol/l) for 2 days. The cell survival was measured using MTT assay. **(b) **MCF10A cells with varying NHERF1 and PTEN levels through retroviral over-expression (NHERF1) or siRNA knockdown (PTEN-KD) were treated with 3 μmol/l STI-571 for 2 days before MTT assay. The reading of untreated control cells was arbitrarily set at 100%. Data from three to five independent experiments are presented (**P < 0.01).

To assess whether PTEN is required for the increased sensitivity to STI-571, we first determined whether altered NHERF1 expression level affected cell responses to STI-571 in a *PTEN*-null background. MDA-MB-468 cells are NHERF1 positive but lack PTEN as a result of homozygous deletion. We knocked down NHERF1 expression in MDA-MB-468 cells using siRNA retrovirus, and these cells were tested for STI-571 sensitivity. We found that in the absence of PTEN the NHERF1-knockdown cells responded to STI-571 at a level similar to that in Babe control cells (Figure 7a). We then determined whether PTEN knockdown was able to influence NHERF1-inducible sensitivity to STI-571 in MCF10A cells. NHERF1 expression (or Neo control) in MCF10A cells was retrovirally transduced and the resultant cells were subjected to PTEN knockdown through retrovirally delivered siRNA (or Babe control). These steps yielded four cell lines with varying PTEN and NHERF1 expression. These cells were then tested for STI-571 sensitivity. PTEN knockdown resulted in a modest increase in proliferation as compared with the control. As expected, over-expression of NHERF1 in parental cells elevated STI-571-inducible killing. In contrast, NHERF1 expression in cells with PTEN knockdown remained highly resistant to STI-571. These findings indicated that increased sensitivity to PDGFR inhibition by NHERF1 was dependent on PTEN (Figure 7b).

### LOH is correlated with lowered NHERF1 expression and wild-type *PTEN *or *PI3KCA*

To explore whether LOH is responsible for a decrease in NHERF1 expression, we correlated the two parameters in 22 breast cancer cell lines that had been tested [15,25]. Among the six cell lines with a high NHERF1 expression level, five (83%) were found to be LOH negative. In contrast, of the 16 cell lines with low and no NHERF1 expression, only two (12.5%) were LOH negative. Thus, LOH at the *NHERF1 *locus is highly associated with lowered NHERF1 expression (*P *= 6.8 × 10^-6^, by Fisher's exact test).

The physical and functional relation between NHERF1 and PTEN indicates they may exist in a common tumor suppressor pathway, which would predict segregation between *NHERF1 *and *PTEN *(or *PI3KCA*) alterations. We thus evaluated whether *NHERF1 *LOH is correlated with *PTEN*/*PI3KCA *mutational status. A total of 39 breast cancer cell lines with available genetic status were used for the correlative analyses [37]. Among the 23 *NHERF1 *LOH-positive cell lines, 15 (65%) had an unaltered *PTEN *or *PI3KCA *gene. In contrast, out of the 16 LOH-negative cell lines, only 4 (25%) showed wild-type *PTEN*/*PI3KCA *status. The genetic alteration in either *PTEN *or *PI3KCA *gene was strongly associated with intact *NHERF1 *alleles (*P *= 0.022, by Fisher's exact test).

## Discussion

Our earlier results of *NHERF1*genetic alterations in human breast cancer prompted us to hypothesize that *NHERF1 *acts as a tumor suppressor gene in mammary gland [15]. Human *NHERF1 *is an estrogen-inducible gene [36]. Estrogen response element half-sites have been located in the 5'-regulatory region of *NHERF1 *gene that are responsible for estrogen-stimulated expression [38]. Because estrogenic signaling is thought to be growth promoting in breast cancer [39], the suppressor activity of NHERF1 was somewhat unexpected. Although the roles of estrogenic induction in NHERF1 biology is not clear, we identified increased growth of breast cancer cells after NHERF1 knockdown [25], which is in agreement with the proposed tumor suppressor function of *NHERF1*. Here we provide additional evidence to support our overall hypothesis.

In breast cells, NHERF1 expression accelerated the turnover of p-Akt induced by PDGF stimulation. This finding was obtained in both over-expression and knockdown models, from both immortalized normal mammary epithelial cells (MCF10A) and a breast cancer line (Zr75.1). The effect of NHERF1 to stimulate the decay of p-Akt is probably through PTEN recruitment by NHERF1 to the cytoplasmic membrane compartment, where active phosphorylation and dephosphorylation of Akt occur. The response is probably related to normal mammary biology, as indicated by the markedly increased levels of p-Akt in the mammary gland tissues of *NHERF1*^-/- ^mice (Figure 4). Elevated p-Akt resulting from *NHERF1 *deletion presumably potentiates the cell survival pathway in mammary gland, where balanced survival and apoptotic signaling is essential for normal development and homeostasis [40]. A deregulated apoptotic process, which leads to defective structural organization and remodeling in mammary gland, is believed to be directly related to breast cancer etiology [41,42]. Interestingly, we recently found that, in contrast to the wild-type mice, *NHERF1*^-/- ^mice exhibited increased ductal side branching and extensive alveolar hyperplasia in mammary gland (our unpublished data). Whether aberrant Akt activation is directly related to hyperplastic morphology in mammary gland warrants further investigation. Also remaining to be established is whether this impaired mammary development is sufficient to increase or accelerate the incidence of mammary tumor.

Because of the strong correlation between *NHERF1 *LOH and the aggressive features of breast cancer, we hypothesized that NHERF1 tumor suppressor activity was haploinsufficient [15]. A number of tumor suppressor genes have been shown to have haploinsufficient activities [43-46]. In these cases, mice carrying an inactivated allele are predisposed to tumor development, and the resulting tumors frequently retain a functional wild-type allele. Interestingly, two NHERF1-binding partners, namely PTEN and NF2, use this mechanism [43,44]. A lowered expression as a result of single allele deletion of the *NHERF1 *gene is obligatory for its haploinsufficient biology. In support of this mechanism, we found that LOH of *NHERF1 *locus in 22 breast cancer cell lines was strongly correlated with lowered NHERF1 protein level. The haploinsufficient expression of *NHERF1 *is also supported by *in vivo *observations. Monoallelic deletion of *NHERF1 *was shown to decrease NHERF1 expression in kidney epithelial cells [31]. Similarly, deletion of one allele of the *NHERF1 *gene resulted in decreased NHERF1 protein expression in mammary gland (Figure 4), providing a plausible link between altered protein expression level and the resultant phenotypic responses. We found lowered NHERF1 expression to be accompanied by a modest increase in p-Akt in the mammary tissue of *NHERF1*^+/- ^mice as compared with that in wild-type (Figure 4). Coincidentally, the mammary glands of *NHERF1*^+/- ^mice exhibited alveolar hyperplasia, albeit less extensively than the *NHERF1*^-/- ^mice (our unpublished data). The correlation suggested an association between abnormal Akt activation and mammary hyperplasia, highlighting the biochemical and pathological consequences of monoallelic deletion of *NHERF1*.

Our findings clearly indicated a dosage effect of *NHERF1 *activity during normal mammary gland development. Whether *NHERF1 *affects breast cancer susceptibility through the haploinsufficiency mechanism requires further investigation. Haploinsuficient *NHERF1 *tumor suppressor activity would also explain the relatively low frequency of intragenic mutations, although the possibility that other NHERF1 pathway components are genetically altered cannot be ruled out.

The present study verifies that NHERF1 has tumor suppressor activity, providing evidence to suggest that its function relies on an intact PTEN pathway. First, NHERF1 is associated with accelerated dephosphorylation of p-Akt, presumably through recruitment of PTEN by NHERF1. Second, knockdown of PTEN abolishes NHERF1-induced sensitivity to chemo-agents. If NHERF1 activity is dependent on PTEN, as our functional study had suggested, then intact *NHERF1 *should be associated with altered *PTEN *(or *PI3KCA*) gene in breast cancer. Our data from 39 breast cancer cell lines showed that this was indeed the case. Collectively, our present study indicates that NHERF1 binds to PTEN to downregulate the PI3K-Akt pathway to elicit tumor suppressor activity. Given that PTEN-PI3K-Akt is one of the most prominent pathways relevant to tumorigenesis and targeted therapy of almost all types of carcinoma, studies on NHERF1 should be instrumental to the development of new strategies to overcome chemo-resistance and enhance efficacy.

In this study we also present evidence that NHERF1 expression status significantly affects how cells respond to PDGFR inhibition. PDGF is among the key growth factors and cytokines that breast cancer cells produce via autocrine or paracrine mechanisms that contribute to malignant progression [47]. Activated PDGF signaling has been shown to prevent cells from undergoing apoptosis during epithelial mesenchymal transition and thus promote breast cancer progression and metastasis [48,49]. As a potent PDGFR inhibitor, STI-571 has been shown to inhibit breast cancer bone metastasis in mouse models [50], and it is being tested clinically in treatment of metastatic breast cancer, among other cancer types [51]. Although the exact mechanism responsible for improved susceptibility to STI-571 by NHERF1 needs further investigation, our present study indicates an inhibitory effect of NHERF1 on PDGF-medicated breast cancer progression and suggests that the status of NHERF1 expression in breast tumor influences how patients respond to STI-571. Because a majority of breast tumors lose NHERF1 expression, our present study raises a possibility of enhancing chemosensitivity by restoring NHERF1 expression. NHERF1 expression may also be used as a biomarker to predict the effectiveness of such treatment.

## Conclusion

In this report, we show that, by interacting with PTEN, NHERF1 accelerates the turnover of p-Akt and enhances the cell sensitivity to STI-571. That NHERF1 elicits suppressor function through PTEN is also indicated by an inverse correlation between intact *NHERF1 *gene and wild-type *PTEN *or *PI3KCA *in breast cancer cells. Given the critical roles of Akt in cell survival and tumorigenesis, the negative regulatory effect of NHERF1 on Akt activity is highly relevant to NHERF1 mammary tumor suppressor function. Our finding of a higher sensitivity of breast cells to STI-571 in the presence of NHERF1 suggests that future investigations of this important pathway may yield new measures to improve breast cancer treatment.

## Abbreviations

ERM = ezrin-radixin-moesin; GST = glutathione S-transferase; LOH = loss of heterozygosity; MEF = mouse embryonic fibroblast; MTT = 3-(4,5-dimethylthiazol-2-yl)-2,5-diphenyltetrazolium bromide; NHERF = Na^+^/H^+ ^exchanger regulatory factor; p-Akt = phospho-Akt; PCR = polymerase chain reaction; PDGFR = platelet-derived growth factor receptor; PI3K = phosphoinositide-3 kinase; PTEN = phosphatase and tensin homolog; siRNA = small interfering RNA.

## Competing interests

The authors declare that they have no competing interests.

## Authors' contributions

YP designed the experiments, carried out technical procedures, and interpreted the data. EJW provided the knockout animals and edited the manuscript. JLD designed the experiments, interpreted the results and wrote the manuscript. All authors approved the final manuscript.
